# Quality Characteristics and Antioxidant Potential of Lemon (*Citrus limon* Burm. f.) Seed Oil Extracted by Different Methods

**DOI:** 10.3389/fnut.2021.644406

**Published:** 2021-09-09

**Authors:** Yong-Sung Park, Il-doo Kim, Sanjeev Kumar Dhungana, Eun-Jung Park, Jae-Jung Park, Jeong-Ho Kim, Dong-Hyun Shin

**Affiliations:** ^1^School of Applied Biosciences, Kyungpook National University, Daegu, South Korea; ^2^Department of International Studies, International Institute of Agricultural Research and Development, Kyungpook National University, Daegu, South Korea; ^3^National Institute of Crop Science, Rural Development Administration, Miryang, South Korea; ^4^Department of Green Technology Convergence, Konkuk University, Chungju-si, South Korea

**Keywords:** antioxidant, free radical scavenger, nutrition, nutraceutical, lemon seed oil

## Abstract

Lemon (*Citrus limon* Burm. f.) is one of the most widely produced and consumed fruits in the world. The seeds of lemon are generally discarded as waste. The purpose of this study was to investigate the quality characteristics and antioxidant potential of lemon seed oil obtained by four extraction methods (roasted-pressing at 170°C, RP-170; roasted-pressing at 100°C, RP-100; cold-pressing, CP; and supercritical fluid, SF). No significant differences in the viscosity, density, and refractive index were observed in the oil obtained from different methods. In the case of Hunter's value, *L* (lightness) and *b* (yellowness) values of SF were higher than those of the others. The oil obtained by the CP method exhibited higher levels of Ca (252.17 mg/kg), Cu (2.38 mg/kg), K (225.98 mg/kg), and Mo (0.47 mg/kg) than that of other methods. The highest contents of total phenols (165.90 mg/mL) and flavonoids (21.69 mg/mL) were significantly high in oil obtained by the SF method. Oleic and linoleic acids consisted of principal fatty acids, which were significantly higher in oil obtained by RP-170. Higher amounts of volatile flavor compounds, such as γ-terpinene, sabinene, and limonene, were observed in CP compared to those observed for the other methods. This study elucidates the effects of different methods of oil extraction on the composition of lemon seed oil and highlights potential applications of these benefits in the food, cosmetic, pharmaceutical, and/or fragrance industries.

## Introduction

Citrus fruits belong to a group of fruits that have notable socio-economic and cultural significance in our society and thus are in high demand (1). There are different kinds of fruits in the citrus group, and lemon (*Citrus limon* Burm. f.) is one of the most important species after orange and mandarin (2). Lemon is a preferred fruit because of its unique flavor and acidity, which make it a value-added food product, and its utility in industrial applications.

Lemon fruit contains various important natural compounds, including phenolics (mainly flavonoids), vitamins, minerals, dietary fiber, essential oils, and carotenoids (3). The health-promoting effects of lemon are mainly associated with vitamin C and flavonoids, which give provide natural antioxidant characteristics (4–6).

The whole citrus fruit plant, including crude extracts of leaves, peels, seeds, and flowers, possesses anticancer activities and antimicrobial potential (7). The fruits are used primarily in juice-processing industries while seeds and peels are generally discarded as waste. During citrus fruit processing for juice, the peels, and seeds as primary byproducts are rich sources of flavonoids that are rare in other plants (8–10). Moreover, it has been reported that citrus processing wastes are abundant and undervalued bio-sources with potential pollution problems (11, 12).

A report shows that citrus seeds contain large amounts of flavonoids, limonoids, and dietary fiber and represent an important source of nutraceutical bioactive compounds (13). The anti-proliferative and anti-aromatase properties of limonoids extracted from lemon seeds are effective against breast cancer cells in humans. The results of photodynamic therapy showed that the efficiency of methylene blue in killing cancer cells increases in the combination with salicylic acid extracted from lemon seeds (14). The lemon seed oil has been used in biodiesel production (15). Most of the lemon oil is obtained from lemon peel by the cold-pressing method and is sold on the market (16). There have several studies related to lemons, but limited studies have been conducted on the yield and quality of lemon seed oil extracted by adopting different methods. Guneser and Yilmaz (17) describe the properties of lemon seed oils extracted by cold-pressed and hexane extraction techniques. The objective of this study was to investigate the physicochemical characteristics, nutrients, volatile compound content, and antioxidant potential of lemon seed oil extracted by using four different techniques. This study may provide an insight into the economic benefits for citrus growers and citrus processing industries.

## Materials and Methods

### Seed Materials

Lemon (*Citrus limon* Burm. f.) seeds were purchased from a supplier (Anhui Highkey Import & Export Co., Anhui, China). Five kilograms of intact seeds were thoroughly washed with tap water and were sun-dried.

### Seed Oil Extraction and Yield Calculation

Lemon seed oil was extracted by four different techniques, and the oil samples were as follows: RP-170, a roasted-pressing extraction that involves oil extraction after roasting and heat pressing at 170°C using an oil extractor (Dongbang machine, DB-500, S. Korea); RP-100: oil extraction after roasting and heat pressing at 100°C using an oil extractor (Wayvin, MA-S1, China); CP, a cold-pressing extraction method that involves oil extraction at room temperature using an oil extractor (Wayvin, MA-S1, China) without pre-roasting of lemon seeds; and SF, a supercritical fluid extraction that involves oil extraction at 400 bar pressure using a CO_2_ extraction system (Ilshin Autoclave, ISA-SEFE-0500-0700-080, Korea) without pre-roasting the seeds. The oil samples extracted using the four different methods were not subjected to any further purification so as to compare their original characteristics. The oil yield was calculated as the ratio of the weight of oil and the weight of the seed used.

### Determination of the Peroxide Value

The peroxide value was determined based on the AOCS method (18). One milliliter of sample oil was put into an Erlenmeyer flask with a 250 mL stopper, and 15 mL acetic acid (HOAc) and 10 mL chloroform (CHCI_3_) were mixed at a ratio of 3:2 followed by continuous stirring until the solution clarified. After adding 0.5 mL of 30% saturated potassium iodide (KI), the stopper was closed immediately and shaken, then left in the dark for 10 min. After 10 min, 25 mL distilled water was added to the solution, the stopper was closed, and the solution was stirred. Then, 1 mL iodine was added and titrated with 0.01 N Na_2_S_2_O_3_ (sodium thiosulfate) solution until the blue reaction mixture changed to a colorless solution. The values were calculated using the following formula:

$$\begin{array}{l} \text{Peroxide value }\left(\text{meq}/\text{kg}\right)=\left(\text{A}-\text{B}\right)\times 0.01\times \text{F}/\text{S}\times 1000 \end{array}$$

where A is the optimum consumption (mL) of the 0.1 N Na_2_S_2_O_3_ sample solution after storage; B is the appropriate consumption (mL) of the 0.1 N Na_2_S_2_O_3_ sample solution at the beginning of the experiment; F is the factor of 0.01 N Na_2_S_2_O_3_; and S is the amount of sample (g).

### Determination of the Acid Value

The acid value was measured as follows. Five grams of sample was put in an Erlenmeyer flask with a stopper and dissolved in the 100 mL ethanol and ether mixture (1:2). The phenolphthalein solution was used as an indicator and titrated with the 0.1 N potassium hydroxide (KOH)-ethanol solution until a pale-red color persisted for 30 s. The acid value was expressed as milligrams of KOH needed to neutralize the free fatty acids contained in 1 g of oil sample as shown below.

$$\begin{array}{l} \text{Acid value }\left(\text{mgKOH}/\text{g}\right)=\left(5.611\times \text{A}\times \text{f}\right)/\text{S} \end{array}$$

where S is the weight of the oil sample (g); A is the consumption (mL) of the 0.1 N KOH solution used in titration; and f is the factor of 0.1 N KOH-ethanol standard solution.

### Physicochemical Properties of Lemon Seed Oil

#### Determination of Viscosity

Lemon seed oil was diluted 10 times with distilled water and homogenized with a homogenizer (T25 Basic, IKA, Germany) at 10,000 rpm for 1 min. In the following procedure, a spindle no. 18 was attached to a viscometer (LVDV-II + Pro, Brookfield Co., Stoughton, MA, USA), and the viscosity of the oil sample was measured at 50 rpm at 25°C. The spindle immersed in the sample was mobilized by the viscometer. The rotation of the spindle caused a viscous drag of the sample, which was measured by the deflection of the calibrated spring. The viscometer was calibrated with the calibration fluid provided by the instrument supplier. The handling of the viscometer and data collection was performed following the manufacturer's instructions.

#### Determination of Density

The density of the oil sample was measured using an electronic densimeter (MD-300S, Alfa Mirage, Tokyo, Japan) at room temperature (25°C). The sample was filled into the density meter cell and degassed prior to measuring the density.

$$\begin{array}{l} \text{ϕ}={\text{ρ}}_{\text{dg}}/{\text{ρ}}_{\text{g}} \end{array}$$

where ρ_dg_ and ρ_g_ are the densities of the degassed and gassed samples, respectively.

#### Determination of the Refractive Index

The refractive index of oil samples was measured using an Abbe refractometer (Atago Co., Ltd., dtm-1, Japan) at room temperature (25°C) with an operating wavelength of 589 nm. A drop of lemon seed oil was placed between the measuring lens and cover plate, and the refractive index was measured.

### Determination of the Color Value

#### Determination of Hunter's Color

The *L* value (lightness), *a* value (redness, + or greenness, –), and *b* value (yellowness, + or blueness, –) of the samples were measured using a colorimeter (Ultra Scan Pro Spectrophotometer, Hunter Lab Inc., Reston, Virginia, USA). Experiments were repeated three times, and the results were expressed as an average value. The *L, a*, and *b* values of the standard white plate were *L* = 99.26, *a* = −0.14, and *b* = −0.16, respectively. The color values of the sample were calculated using Hunter *L, a*, and *b* values and those of the standard plate.

#### Determination of Browning Color

One hundred milliliters of distilled water was added to 20 g of the sample and homogenized at 3,000 rpm for 1 min using a homogenizer (Nissei AM-11, Nihonseiki Kaisha Ltd., Japan). The brownness of the sample was measured using a UV-Visible spectrophotometer (UV Spectrophotometer 1601, Shimadzu, Kyoto, Japan) at 645 nm.

### Determination of the Mineral Content

The mineral content of lemon seed oil was determined according to the Association of Official Agricultural Chemists (AOAC) method (19). One milliliter sample was added in 30 mL 2.0 N HNO_3_ and was left at room temperature for 12 h, followed by heating at 100°C. Twenty milliliters of 2.0 N HNO_3_ was added to the solution and filtered through a 0.45 μm membrane filter. Mineral elements Ca, Co, Cu, Fe, K, Mg, Mn, Mo, Na, and Zn were analyzed by an inductively coupled plasma-atomic emission spectrometer (ICP-AES). The analysis conditions were as follows: approximate 1,150 W RF power, 100 rpm pump rate, 10.5–15 L/min plasma gas flow rate, 1.5 L/min auxiliary gas flow rate, 20 psi nebulizer pressure, 15 mm observation height, and 1–5 s copy and reading time.

### Antioxidant Determination

#### Determination of DPPH Free Radical Scavenging Potential

The 1,1-diphenyl-2-picryl-hydrazyl (DPPH) radical scavenging activity was measured by slightly modifying the method of Blois (20). First, a 0.1% concentration of methanolic DPPH solution was prepared for this experiment. The DPPH solution and methanol 1:1 (C), methanol (Co), DPPH solution and sample 1:1 (S), and methanol and sample 1:1 (So) were mixed in 96-well plates and incubated for 30 min under dark conditions. The absorbance value of the reaction mixtures was measured at 517 nm using a microplate spectrophotometer (Multiskan GO; Thermo Fisher Scientific Oy, Vantaa, Finland). The DPPH free radical scavenging potential was calculated by the following equation,

$$\begin{array}{l} \text{Scavenging activity}\left(\text{\%}\right)=\left[1-\left(\text{S}-\text{So}\right)/\left(\text{C}-\text{Co}\right)\right]\times 100 \end{array}$$

#### Determination of the Total Polyphenol Content

The total polyphenol content of the oil samples was determined using the Folin–Ciocalteu method (21). A 50 μL of the sample was added to 250 μL of 1 N Folin–Ciocalteu reagent. After 1 min, 750 μL of 20% (w/v) aqueous Na_2_CO_3_ was added, and the volume increased to 1.0 mL by adding distilled water and was incubated at room temperature under dark conditions. After incubation for 2 h, the absorbance was measured at 750 nm using a spectrophotometer (Multiskan GO, Thermo Fisher Scientific). The total phenol contents were calculated as the equivalent of gallic acid (μg GAE/ mL), which was used to plot the standard calibration curve. The concentrations of the standard used to plot the calibration plot were 62.5, 125, 250, 500, and 1,000 ppm (Supplementary Figure 1).

#### Determination of the Total Flavonoid Content

The total flavonoid content of the sample was determined by following the method of Michalska et al. (22). Fifty microliters of samples and 250 μL of 5% NaNO_2_ (w/v) were mixed. The reaction mixtures were incubated at room temperature for 6 min. Subsequently, 300 μL of 10% AlCl_3_ (w/v) solution was added to the reaction mixtures and incubated at room temperature for 5 min. One milliliter of 1 M NaOH was added to the mixture and mixed well using a vortexer. A UV spectrophotometer (Shimadzu Co., Kyoto, Japan) was used to read the absorbance values of the reaction mixtures at 510 nm. The standard curve was plotted using quercetin (QE) diluted at different concentrations (62.5, 125, 250, 500, and 1,000 ppm) (Supplementary Figure 2).

### Determination of the Fatty Acid Content

The fatty acid analysis was performed following the method described by Malacrida et al. (23). Twenty-five milligrams of the sample and 1 mL of the internal standard solution were placed into a glass tube. Then, 1.5 mL of 0.5 N methanolic NaOH solution was added to the mixture and heated at 100°C using a heating block for 5 min. After cooling, 2 mL of 14% boron trifluoride-methanol solution was added to the mixture. The mixture was heated at 100°C for 30 min. After cooling the mixture to 30–40°C, 1 mL isooctane solution was added and shaken for 30 s. Immediately, 5 mL saturated sodium chloride was added and shaken. After the sample was cooled to room temperature, the water layer and isooctane layer were separated. The isooctane layer was dehydrated with anhydrous sodium sulfate and used as a test solution. The solution was analyzed for the fatty acid content using a gas chromatograph (GC) (7890A, Agilent Technology, Palo Alto, CA, USA). The GC conditions for fatty acid analysis were as follows: 100 m 0.25 mm 0.20 m SP-2560 capillary column (Supelco, Bellefonte, PA, USA), 125–240°C oven temperature (125°C for 4 min, 125–170°C for 5 min, 180–210°C for 20 min, 210–240°C for 15 min), 240°C injection temperature, 250°C detector temperature, 1 L injection volume, and 1.2 mL/min flow rate.

### Determination of Volatile Flavor Compounds

The volatile compounds of lemon seed oil samples were extracted by headspace- solid phase microextraction (HS-SPME) and analyzed using a gas chromatography/mass spectrometry (GC/MS) technique by following the method described earlier (24). Two hundred milliliters of 3-octanol was put into 5 mL of the sample, and 5 g sodium sulfate was added into the mixture. The volatile compounds of the samples were extracted by solid-phase microextraction (SPME) fiber (50/30-μm DVB/CAR/PDMS Supelco, Bellefonte, PA, USA) after shaking the mixture at 40°C for 30 min on a heating platform agitation at 400 rpm. The pre-conditioned (according to the manufacturer's instruction) SPME was inserted into the headspace, where extraction was carried out for 30 min under continuous heating and agitation by a magnetic stirrer. The fiber was then desorbed into the GC injector for 25 min.

The GC/MS analysis was carried out using an Agilent 7890B GC (Agilent Technologies Inc., Santa Clara, CA, USA) system coupled to an Agilent MSD 5977B quadruple mass spectrometer (Agilent Technologies Inc., Santa Clara, CA, USA). The capillary column used was of 60 m length, 0.25 μm i.d., 0.25 μm film thickness (DB-WAX, Agilent Technologies, Middleburg, OI, USA). The sample was injected by placing the SPME fiber at the GC inlet for 25 min with the splitless mode. The oven temperature of the GC was initially maintained at 40°C for 2 min, then increased to 220°C at the rate of 3°C/min and held at 220°C for 10 min. Helium was used as the carrier gas at a constant flow rate of 1 mL/min. A series of n alkanes (C_6_-C_22_) was analyzed under the same conditions to obtain the Linear Retention Index values for the reaction mixture components. In full-scan mode, the electron ionization mass spectra in the range of 35–350 m/z were recorded at 70 eV electron energy. The mass spectrometer was operated in electron impact mode with an electron energy of 70 eV and an emission current of 50 μA and scanned from m/z 28 to 450 at 1.7 scans/s. The mass spectra were obtained in full-scan mode and compared with the Wiley 275 mass spectral database (Agilent Technologies, Santa Clara, CA, USA).

### Statistical Analysis

Data were subjected to an analysis of variance using SAS 9.4. Differences between the means at *p* < 0.05 were identified using Tukey's test. The average value of three replicates was reported unless otherwise mentioned.

## Results

### Yield, Peroxide Value, and Acid Value of Lemon Seed Oil

The yield, peroxide value, and acid value of the lemon seed oil extracted by different methods are shown in Table 1. The extraction yields for RP-170, RP-100, CP, and SF were 29.28, 26.65, 35.85, and 32.08%, respectively. The AVs of the oil samples were in the range of 1.75–2.70. The peroxide value was in the range of 13.72–27.41 in all treatments.

**Table 1:** Yield, peroxide value (POV), and acid value (AV) of lemon seed oil extracted using different methods.

**Particular**	**Sample^1^**			
	**RP-170**	**RP-100**	**CP**	**SF**
Yield (%)	29.28 ± 2.02^bc^^2^	26.65 ± 1.65^c^	35.85 ± 0.85^a^	32.08 ± 1.45^b^
POV(meq/kg)	16.87 ± 0.04^b^	13.72 ± 0.44^d^	14.51 ± 0.57^c^	27.41 ± 0.78^a^
AV(mg KOH/g)	2.46 ± 0.07^c^	2.58 ± 0.13^b^	2.70 ± 0.65^a^	1.75 ± 0.09^d^

1 *RP-170, oil extraction after roasting and heat pressing at 170°C; RP-100, oil extraction after roasting and heat pressing at 100°C; CP, cold-pressing extraction; and SF, supercritical fluid extraction*.

2 *Quoted values are means ± SD of triplicate measurements. Value followed by different superscripts in the same row are significantly different (p < 0.05)*.

### Physicochemical Properties of Lemon Seed Oil

The physicochemical properties of lemon seed oil, which are shown in Table 2, play an important role in oil formation and stability. The refractive index of lemon seed oil was in the range of 1.470–1.471, which was almost the same in different samples. The density of lemon seed oil was highest in SF, followed by that in RP-170, and was lowest in RP-100 and CP. The viscosity of lemon seed oil was 92.67–104.67 cP.

**Table 2:** Physicochemical properties of lemon seed oils extracted using different methods.

**Sample^1^**	**Viscosity (cP)**	**Density (g/cm3)**	**Refractive index**
RP-170	92.67 ± 0.00^d^^2^	0.914 ± 0.001^b^	1.471 ± 0.000^a^
RP-100	102 ± 0.00^b^	0.912 ± 0.001^c^	1.470 ± 0.000^b^
CP	94 ± 1.15^c^	0.912 ± 0.000^c^	1.470 ± 0.000^b^
SF	104.67 ± 1.15^a^	0.920 ± 0.000^a^	1.470 ± 0.000^b^

1 *Samples are defined in Table 1*.

2 *Quoted values are means ± SD of triplicate measurements. Value followed by different superscripts in the same column are significantly different (p < 0.05)*.

### Color and Browning Color Intensity of Lemon Seed Oil

Hunter's color and BCI were indicators of consumer preference. The color values of lemon seed oil produced using four different extraction methods significantly varied (Table 3). The *L*-values of RP-170, RP-100, CP, and SF were 24.54, 25.01, 24.78, and 36.32, respectively. The *a*-values of RP-170, RP-100, CP, and SF were 0.97, 0.61, 1.28, and 1.76, respectively. The *b*-values of RP-170, RP-100, CP, and SF were 1.27, 1.35, 3.11, and 12.94, respectively. The values of the two roasted samples RP-170 and RP-100 were lower than those of the non-roasted samples. The BCI was lowest in SF and highest in RP-100. The BCI values of the four samples were significantly different, with the highest value in RP-100 (2.84) and the lowest value in SF (0.668) (Table 3).

**Table 3:** Color value and browning color intensity of lemon seed oils extracted using different methods.

**Color value^2^**	**Sample^1^**			
	**RP-170**	**RP-100**	**CP**	**SF**
*L* (Lightness)	24.54 ± 0.06^d^^4^	25.01 ± 0.02^b^	24.78 ± 0.03^c^	36.32 ± 0.10^a^
*a* (Redness)	0.97 ± 0.03^c^	0.61 ± 0.02^d^	1.28 ± 0.02^b^	1.76 ± 0.03^a^
*b* (Yellowness)	1.27 ± 0.01^d^	1.35 ± 0.02^c^	3.11 ± 0.03^b^	12.94 ± 0.12^a^
BCI^3^	1.520 ± 0.302^b^	2.840 ± 0.089^a^	1.357 ± 0.316^c^	0.668 ± 0.005^d^

1 *Samples are defined in Table 1*.

2 *L, lightness (100, white; 0, black); a, redness (–, green; +, red); b, yellowness (–, blue; +, yellow)*.

3 *Browning color intensity*.

4 *Quoted values are means ± SD of triplicate measurements. Value followed by different superscripts in the same row are significantly different (p < 0.05)*.

### Mineral Content

The total mineral content of the oil samples was substantially reduced in SF 93.42 (mg/kg) compared to that in other methods (364.65–594.85 mg/kg) of oil extraction (Table 4). Interestingly, the amount of Zn was significantly high in SF among the four methods, and that of Na was higher than in RP-170 and RP-100. Several mineral elements, including Ca, Cu, K, and Mo, were significantly high in CP compared to other methods of oil extraction.

**Table 4:** Mineral contents (mg/kg) of lemon seed oils extracted using different methods.

**Element**	**Sample^1^**			
	**RP-170**	**RP-100**	**CP**	**SF**
Ca	132.47 ± 5.41^c^^2^	220.45 ± 14.98^b^	252.17 ± 13.05^a^	25.61 ± 0.15^d^
Cu	1.60 ± 0.03^c^	0.94 ± 0.01^d^	2.38 ± 0.02^a^	2.27 ± 0.02^b^
Fe	10.51 ± 0.15^d^	20.72 ± 0.30^a^	19.66 ± 0.21^b^	14.83 ± 0.28^c^
K	136.72 ± 8.61^c^	193.36 ± 11.62^b^	225.98 ± 9.972^a^	12.14 ± 14.85^d^
Mg	64.95 ± 3.56^a^	66.29 ± 4.27^a^	69.88 ± 3.08^a^	3.82 ± 0.35^b^
Mn	0.86 ± 0.02^a^	0.80 ± 0.02^b^	0.90 ± 0.03^a^	0.15 ± 0.005^c^
Na	8.57 ± 0.64^c^	10.35 ± 0.72^b^	18.19 ± 0.80^a^	19.92 ± 2.85^a^
Zn	8.83 ± 0.11^c^	11.52 ± 0.12^b^	5.19 ± 0.07^d^	14.56 ± 0.18^a^
Co	0.03 ± 0.003^b^	0.05 ± 0.002^a^	0.03 ± 0.003^b^	0.02 ± 0.002^c^
Mo	0.11 ± 0.004^c^	0.23 ± 0.02^b^	0.47 ± 0.06^a^	0.10 ± 0.02^c^
Total	364.65	524.71	594.85	93.42

1 *Samples are defined in Table 1*.

2 *Quoted values are means ± SD of triplicate measurements. Value followed by different superscripts in the same row are significantly different (p < 0.05)*.

### Antioxidant Potential

The total polyphenol and flavonoid contents and DPPH free radical scavenging potential of oil samples significantly (*p* < 0.05) varied by the method of extraction (Table 5). The highest antioxidant potential of lemon seed oil was found for SF, and the lowest was in RP-170. The total polyphenol and flavonoid contents were significantly high in SF (165.90 μg GAE/mL and 21.69 μg QE/mL), followed by that in RP-100 (145.08 μg GAE/mL and 15.99 μg QE/mL), CP (119.59 μg GAE/mL and 13.23 μg QE/mL), and RP-170 (82.46 μg GAE/mL and 11.88 μg QE/mL).

**Table 5:** DPPH radical scavenging activity and total polyphenol and flavonoid contents of lemon seed oils obtained by different extraction methods.

**Particular**	**Sample^1^**			
	**RP-170**	**RP-100**	**CP**	**SF**
Total polyphenol (μg GAE/mL)	82.46 ± 1.56^d^^2^	145.08 ± 1.72^b^	119.59 ± 0.23^c^	165.90 ± 1.94^a^
	(100%)^3^	(175.94%)	(145.03%)	(201.19%)
DPPH (% Inhibition)	71.1	88.5	75.8	99.2
Flavonoid (μg QE/mL)	11.88 ± 0.19^d^	15.99 ± 0.14^b^	13.23 ± 0.86^c^	21.69 ± 0.82^a^
	(100%)	(134.60%)	(111.36%)	(182.58%)

1 *Samples are defined in Table 1*.

2 *Quoted values are means ± SD of triplicate measurements. Value followed by different superscripts in the same row are significantly different (p < 0.05)*.

3 *Values in parentheses represent the relative percentage of lemon seed oil extracted by the RP-170 method*.

### Fatty Acid Content of Lemon Seed Oil by Different Extraction Methods

The fatty acid composition of oil samples obtained from different methods of oil extractions was significantly different (Table 6). In particular, the contents of five major fatty acids, C16:0, C18:0, C18:1n9c, C18:2n6c, and C18:3n3, were higher than the contents of other fatty acids. The contents of saturated fatty acids C16:0 and C18:0 reached a high value using the RP-100 (16.868 g/100 g) and CP (3.412 g/100 g) methods. The contents of unsaturated fatty acids in the lemon seed oil samples were higher than those of saturated fatty acids.

**Table 6:** Fatty acid content of lemon seed oils extracted using different extraction methods (g/100 g).

**Fatty acid**	**Sample^1^**			
	**RP-170**	**RP-100**	**CP**	**SF**
**Saturated fatty acid**				
C4:0 (Butyric acid)	ND^2^	ND	ND	ND
C6:0 (Caproic acid)	0.003^3^	ND	ND	ND
C8:0 (Caprylic acid)	0.004^a^	0.002^a^	ND	ND
C10:0 (Capric acid)	0.001^a^	0.001^a^	ND	ND
C11:0 (Undecylic acid)	ND	ND	ND	ND
C12:0 (Lauric acid)	0.004^a^	0.003^a^	0.003^a^	0.004^a^
C13:0 (Tridecylic acid)	ND	ND	ND	ND
C14:0 (Myristic acid)	0.050^a^	0.043^ab^	0.039^b^	0.033^b^
C15:0 (Pentadecylic acid)	0.020^a^	0.022^a^	0.022^a^	0.016^a^
C16:0 (Palmitic acid)	16.621^a^	16.868^a^	15.749^a^	11.680^b^
C17:0 (Margaric acid)	0.157^a^	0.170^a^	0.169^a^	0.124^b^
C18:0 (Stearic acid)	2.897^b^	2.880^b^	3.412^a^	1.955^c^
C20:0 (Arachidic acid)	0.257^a^	0.257^a^	0.286^a^	0.162^b^
C21:0 (Heneicosylic acid)	0.004^a^	0.005^a^	ND	ND
C22:0 (Behenic acid)	0.072^a^	0.078^a^	0.081^a^	0.067^a^
C23:0 (Tricosylic acid)	0.016^a^	0.017^a^	0.017^a^	0.013^a^
C24:0 (Lignoceric acid)	ND	ND	ND	ND
Sub-total	20.106	20.346	19.778	14.054
**Monounsaturated fatty acid**				
C14:1 (Myristoleic acid)	ND	ND	ND	ND
C15:1 (cis-10-Pentadecenoic acid)	ND	ND	ND	ND
C16:1 (Palmitoleic acid)	0.507^a^	0.510^a^	0.388^b^	0.302^a^
C17:1 (cis-10-Heptadecenoic acid)	0.114^a^	0.103^a^	0.071^a^	0.044^a^
C18:1n9c (Oleic acid)	18.653^a^	18.530^a^	16.033^a^	11.107^b^
C18:1n9t (Elaidic acid)	0.005^c^	0.003^c^	0.178^a^	0.122^b^
C20:1 (cis-11-Eicosenoic acid)	ND	ND	0.069^a^	0.038^a^
C22:1n9 (Erucic acid)	ND	ND	ND	ND
C24:1 (Nervonic acid)	ND	ND	ND	ND
Sub-total	19.279	19.146	16.739	11.613
**Polyunsaturated fatty acid**				
C18:2n6t (Linolelaidic acid)	0.028^a^	0.032^a^	0.038^a^	0.037^a^
C18:2n6c (Linoleic acid)	26.265^a^	27.039^a^	23.652^a^	15.510^b^
C18:3n6 [Linolenic (GLA) acid]	ND	ND	0.006	ND
C18:3n3 [Linolenic (ALA) acid]	5.542^a^	2.801^b^	2.104^b^	1.507^b^
C20:2 (cis-11,14-Eicosadienoic acid)	0.015^b^	0.013^b^	0.379^a^	0.492^a^
C20:3n3 (cis-11,14,17-Eicosatrienoic acid)	ND	ND	ND	ND
C20:3n6 (cis-8,11,14-Eicosatrienoic acid)	0.005^b^	0.005^b^	0.151^a^	0.178^a^
C20:4n6 (Arachidonic acid)	ND	ND	ND	ND
C20:5n3 (Eicosapentaenoic acid)	0.062^a^	0.063^a^	0.065^a^	0.041^b^
C22:2 (cis-13,16-Docosadienoic acid)	ND	ND	0.003	ND
C22:6n3 (cis-4,7,10,13,16,19- Docosahexaenoic acid)	ND	ND	ND	ND
Sub-total	31.917	29.953	26.398	17.765
Total	71.302	69.445	62.915	43.432

1 *Samples are defined in Table 1*.

2 *ND, not detectable*.

3 *Quoted values are means of duplicate measurements. Value followed by different superscripts in the same row are significantly different (p < 0.05)*.

### Volatile Flavor Content

The profiles of the volatile flavor compounds detected in the oil samples are shown in Table 7. The major constituent in the samples was monoterpene substances. The contents of γ-terpinene, α-pinene, sabinene, β-pinene, and β-myrcene (1.97, 0.17, 0.77, 0.18, and 0.71 peak area %) in lemon seed oil obtained in CP were higher than in other methods. The limonene content was high in RP-170 (27.39 peak area %) and CP (27.24 peak area %) as compared to that of the other methods (Supplementary Figures 3–6).

**Table 7:** Volatile compounds of lemon seed oil extracted using different extraction methods (Unit: peak area %).

**Compounds**	**Sample^1^**			
	**RP-170**	**RP-100**	**CP**	**SF**
γ-terpinene	1.33^a^^2^	1.46^a^	1.97^a^	1.04^a^
α-pinene	0.07^b^	0.07^b^	0.17^a^	0.20^a^
Sabinene	ND^3^	ND	0.77	ND
β-pinene	ND	ND	0.18	ND
β-myrcene	0.52^b^	0.29^c^	0.71^a^	ND
Limonene	27.39^a^	15.63^b^	27.24^a^	23.25^a^
Decane	0.29^c^	2.35^b^	3.70^a^	0.32^c^
2,5-Dimethyl-nonane	ND	0.33^a^	0.51^a^	ND
4-Methyl-decane	ND	0.54^a^	0.82^a^	ND
Hexanal	4.14^a^	1.89^c^	2.87^b^	0.41^d^
Dodecane	0.95^b^	2.03^a^	2.43^a^	0.53^b^
1-Octen-3-ol	1.36^a^	1.06^a^	0.88^a^	ND
2-Furan-carboxaldehyde	5.45^a^	2.15^b^	0.26^c^	ND
Cyclotrisiloxane, hexamethyl-	ND	1.67^b^	3.42^a^	0.66^c^
Benzaldehyde	ND	2.11^a^	ND	1.15^b^
2,3-Butanediol	1.34^b^	1.86^b^	3.76^a^	3.65^a^
Methyl salicylate	0.48^b^	2.12^a^	2.27^a^	ND
2,4-Decadienal, (E,E)-	3.16^a^	0.21^b^	ND	ND
Benzeneethanol	ND	0.77^b^	1.12^b^	2.61^a^
p-Cymene	ND	ND	1.68	ND
o-Cymene	2.14^a^	ND	ND	1.25^a^
Acetic acid	0.11^b^	ND	0.98^b^	9.09^a^
3,5-Octadien-2-one	1.79^b^	ND	2.97^a^	ND
1H-purine-2,6-dione, 3,7-dihydro-1,3,7-trimethyl-	ND	ND	3.74	ND
β-Selinene	ND	ND	ND	2.94
Octanoic acid	ND	ND	ND	2.44

1 *Samples are defined in Table 1*.

2 *Quoted values are means of duplicate measurements*.

3 *ND, not detectable. Value followed by different superscripts in the same row are significantly different (p < 0.05)*.

## Discussion

Extraction is a major step in seed oil processing because it is highly associated with the quality and quantity of oil extracted. The selection of appropriate oil extraction technique is, therefore, critical for enhancing the yield and quality of the oil. In the present study, we compared the yield and quality of lemon seed oil extracted by four different techniques. It is assumed that if the seeds are roasted for a long time at high temperature, the tissues are broken, and oil will be extracted easily, but it is also known that high heat and a long roasting time destroy the suitability of tissues for extraction and lower the oil yield (25). As a result, the yields of RP-170 and RP-100, in which oil was extracted after the seeds were roasted at high temperature, were lower than those of CP and SF, in which oil was extracted without applying heat. A higher oil yield was also found in rosehip seed extracted with the CP method compared to that of SF method (26). Generally, the acid value of fresh oil is approximately <1.00 (27). The acid value of the oil sample extracted by SF was close to that of the fresh oil, but the other three oil samples exceeded the value. It has been reported that the stability of the peroxide value is increased as the roasting temperature of the sample increases (28). The acid value and peroxide value obtained in the present study were higher than that found in a previous study (29). The refractive index is indicative of the turbidity of the oil (30), which may determine the rancidity of edible oils and fats (31). The increasing density affects the tendency of oil to cream (30). Viscosity is particularly important when considering the utility of oil as a lubricant. The refractive index (1.47) of lemon seed oil extracted using the cold-pressed and solvent extraction methods (29) was comparable with that obtained in the present study.

The lightness and yellowness values of CP oil were comparable with that obtained in the lemon seed oil (29). The difference in the color values and BCI of different samples might be due to the difference in antioxidants (32) and Maillard's reaction (33). The higher BCI of RP-170 and RP-100 might be due to roasting the seeds at high temperatures (34). The amounts of mineral elements such as Ca, K, and Mg that are reported to provide health benefits for the prevention and treatment of essential hypertension (35) were higher in CP. However, Zn and Fe, which are often lacking in the human diet (36), were high in two different samples SF and RP-100, respectively. Overall, the oil extracted using the SF method contained approximately four times fewer total minerals than that of the other three methods. Similarly, the antioxidant potential, measured through DPPH free radical scavenging potential and the total polyphenol and flavonoid content, was significantly high in the SF method. A higher total phenolic content was also observed in the *Nigella sativa* seed oil extracted by SF than CP methods (37). Phenolic compounds are considered to possess antioxidant properties in vegetables and other foods (38, 39), and provide numerous health benefits (40). A considerable amount of mono- and polyunsaturated fatty acids in lemon seed oil indicates the substantial potential of using it as an edible oil (41). The quality of unsaturated fatty acids found in citrus is expected to provide good quality as an edible oil (42). Unsaturated fatty acids are found to be good for human health (43, 44).

To the best of the authors' knowledge, this is the first report on the comparison of volatile flavor compounds of lemon seed oil extracted by different techniques. Guneser and Yilmaz (17) found that the concentration of the majority of aromatic volatile compounds was higher in the lemon seed oil extracted by CP method than in the solvent extraction method. However, there are several reports on the volatile flavor compounds of other parts of the lemon; for instance, peel and leaf (45), flower organs, pollen (46, 47), and peel (48–50). Cannon et al. (48) found more than 150 flavor compounds in lemon peel, including some sulfur monoterpenoids, non-terpenoid esters, and aldehydes, that could not be identified in nine other citrus species (51). Thus, lemon provides a wide variety of special flavor compounds for the food, beverage, cosmetic, pharmaceutical, and fragrance industries. One of the important monoterpenes found in lemon seed oil was limonene. Monoterpenes such as limonene are effective in early cancer treatment, and limonene is currently being evaluated in clinical trials for stage-I cancer patients (52). Lemon seed oil shows potential utility in the food and pharmaceutical industries. Limonene is present in several citrus oils, including lemon (53). Limonene is sweet, has a lemon-like fragrance, and is extensively used as an additive in several industrial products, such as perfumes, soaps, foods, and beverages, and is an alternative of petroleum in cleaning products and paints (54). Lemon seed oil could be potential biodiesel due to possessing essential features of biodiesel, such as viscosity and density that are comparable with the European standard (55). Several physical and chemical properties of lemon seed oil were also studied for their utility as biodiesel (15). Moreover, few biodiesel properties, including acid value, water content, and flash and combustion points, are also highly dependent on the conditions of the production process (56). In the same study, the qualities of biodiesel produced from rapeseed, sunflower, and high oleic sunflower were found to be better than those obtained from soybean, *Cynara cardunculus* L., *Brassica carinata*, and *Jatropha curcas*. Biodiesel fuels are environmentally friendly compared to fossil fuels due to their biodegradability and non-toxic properties, and they provide engine lubricity to low sulfur diesel (57–59).

## Conclusion

Lemon seed oils extracted using four different methods were investigated. A higher amount of total mineral was found in RP-100 and CP, whereas that of Zn was higher in SF. The total polyphenol and flavonoid contents were significantly higher in SF. The polyunsaturated fatty acid content was higher in RP-170. The results showed that the method of oil extraction could significantly affect the physicochemical, nutritional, and antioxidant properties of lemon seed oil. Thus, this study provides insight into the utility of various methods of oil extraction based on their specific use in the food, beverage, cosmetic, pharmaceutical, and/or fragrance industries.

## Data Availability Statement

The raw data supporting the conclusions of this article will be made available by the authors, without undue reservation.

## Author Contributions

Y-SP prepared the literature search, conceptualized the study, and conducted the experiment. E-JP and J-JP conducted the experiment. J-HK, SD, and I-dK conducted the statistical analysis. Y-SP and SD drafted the manuscript. D-HS supervised the study and revised and approved the manuscript. All authors contributed to the article and approved the submitted version.

## Funding

This study was supported by the Research Fund, 2020 of Kyungpook National University, Daegu, Korea.

## Conflict of Interest

The authors declare that the research was conducted in the absence of any commercial or financial relationships that could be construed as a potential conflict of interest.

## Publisher's Note

All claims expressed in this article are solely those of the authors and do not necessarily represent those of their affiliated organizations, or those of the publisher, the editors and the reviewers. Any product that may be evaluated in this article, or claim that may be made by its manufacturer, is not guaranteed or endorsed by the publisher.

## References

[1] Iglesias DJ Cercós M Colmenero-Flores JM Naranjo MA Ríos G Carrera E. Physiology of citrus fruiting. Braz J Plant Physiol. (2007) 19:333–62. 10.1590/S1677-04202007000400006

[2] Perez-Perez JG Castillo IP Garcia-Lidon A Botia P Garcia-Sanchez F. Fino lemon clones compared with the lemon varieties Eureka and Lisbon on two rootstocks in Murcia (Spain). Sci Hortic. (2005) 106:530–8. 10.1016/j.scienta.2005.05.004

[3] González-Molina E Domínguez-Perles R Moreno DA García-Viguera C. Natural bioactive compounds of for food and health. J Pharm Biomed Anal. (2010) 51:327–45. 10.1016/j.jpba.2009.07.027 19748198

[4] Vinson JA Proch J Bose P. Determination of quantity and quality of polyphenol antioxidants in foods and beverages. Methods Enzymol. (2001) 335:103–14. 10.1016/S0076-6879(01)35235-7 11400359

[5] Proteggente AR Pannala AS Paganga G Buren LV Wagner E Wiseman S. The antioxidant activity of regularly consumed fruit and vegetables reflects their phenolic and vitamin C composition. Free Radical Res. (2002) 36:217–33. 10.1080/10715760290006484 11999391

[6] Wilmsen PK Spada DS Salvador M. Antioxidant activity of the flavonoid hesperidin in chemical and biological systems. J Agric Food Chem. (2005) 53:4757–61. 10.1021/jf0502000 15941311

[7] Kawaii S Tomono Y Katase E Ogawa K Yano M Koizumi M. Quantitative study of flavonoids in leaves of citrus plants. J Agric Food Chem. (2000) 48:3865–71. 10.1021/jf000100o 10995283

[8] Bocco A Cuvelier ME Richard H Berset C. Antioxidant activity and phenolic composition of citrus peel and seed extracts. J Agric Food Chem. (1998) 46:2123–29. 10.1021/jf9709562

[9] Mandalari G Bennett RN Bisignano G Saija A Dugo G Lo Curto RB. Characterization of flavonoids and pectins from bergamot (*Citrus bergamia* Risso) peel, a major byproduct of essential oil extraction. J Agric Food Chem. (2006) 54:197–203. 10.1021/jf051847n 16390199

[10] Roy SD Bania R Chakraborty J Goswami R Laila R Ahmed SA. Pharmacognostic, phytochemical, physicochemical property andantimicrobial activity studies of lemon peel oil. J Nat Prod Plant Resour. (2012) 2:431–5.

[11] Russo M Bonaccorsi I Inferrera V Dugo P Mondello L. Underestimated sources of flavonoids, limonoids and dietary fiber: availability in orange's by-products. J Funct Foods. (2015) 12:150–7. 10.1016/j.jff.2014.11.008

[12] Tejada-Ortigoza V Garcia-Amezquita LE Serna-Saldívar SO Welti-Chanes J. Advances in the functional characterization and extraction processes of dietary fiber. J Food Eng Rev. (2016) 8:251–71. 10.1007/s12393-015-9134-y

[13] Kim J Jayaprakasha GK Patil BS. Limonoids and their anti-proliferative and anti-aromatase properties in human breast cancer cells. Food Funct. (2013) 4:258–65. 10.1039/C2FO30209H 23117440

[14] Hosseinzadeh R Khorsandi K Jahanshiri M. Combination photodynamic therapy of human breast cancer using salicylic acid and methylene blue. Spectrochim Acta Part A. (2017) 184:198–203. 10.1016/j.saa.2017.05.008 28499173

[15] Sarno M Ponticorvo E. A new nanohybrid for electrocatalytic biodiesel production from waste Amalfi coast lemon seed oil. Fuel. (2020) 267:117178. 10.1016/j.fuel.2020.117178

[16] Misharina TA Terenina MB Krikunova NI Medvedeva IB. Autooxidation of a mixture of lemon essential oils, methyl linolenoate, and methyl oleinate. Appl Biochem Microbiol. (2010) 6:599–604. 10.1134/S0003683810050157 21061607

[17] Guneser BA Yilmaz E. Bioactives, aromatics and sensory properties of cold-pressed and hexane-extracted lemon (*Citrus limon* L.) seed oils. J Am Oil Chem Soc. (2017) 94:723–31. 10.1007/s11746-017-2977-z

[18] AOCS. Official Method Cd 8-53. Official Method and Tentative Methods of the AOCS. 4th ed. Champaign, IL, American Oil Chemists' Society Press (1996).

[19] AOAC. Official Methods of Analysis. 16th ed. Washington, DC: Association of Official Analytical Chemists (1995).

[20] Blois MS. Antioxidant determinations by the use of a stable free radical. Nature. (1958) 181:1199–200. 10.1038/1811199a0

[21] Singleton VL Orthofer R Lamuela-Raventos RM. Analysis of total phenols and other oxidation substrates and antioxidants by means of folin-ciocalteu reagent. Methods Enzymol. (1999) 299:152–78. 10.1016/S0076-6879(99)99017-1

[22] Michalska A Ceglinska A Amarowicz R Piskula MK Szawara-Nowak D Zielinski H. Antioxidant contents and antioxidative properties of traditional rye breads. J Agric Food Chem. (2007) 55:734–40. 10.1021/jf062425w 17263468

[23] Malacrida CR Kimura M Jorge N. Phytochemicals and antioxidant activity of citrus seed oils. Food Sci Technol Res. (2012) 18:399–404. 10.3136/fstr.18.399

[24] Howard KL Mike JH Riesen R. Validation of a solid-phase microextraction method for headspace analysis of wine aroma components. Am J Enol Vitic. (2005) 56:37–45.

[25] Ha JH Kim DH. Changes in the physico-chemical properties of the meals from the defatted sesame seeds at various roasting temperature and time. Korean J Food Sci Technol. (1996) 28:246–52.

[26] del Valle JM Bello S Thiel J Allen A Chordia L. (2000). Comparison of conventional and supercritical CO_2_-extracted rosehip oil. Braz J Chem Eng. (1996) 17:335–48. 10.1590/S0104-66322000000300010

[27] Xie WQ Chai XS. An efficient method for determining the acid value in edible oils by solvent-assisted headspace gas chromatography. Anal Methods. (2016) 8:5789–93. 10.1039/C6AY01121G

[28] Kim HW. Studies on the antioxidative compounds of sesame oils with roasting temperature. Korean J Food Sci Technol. (2000) 32:246–51.

[29] Yilmaz E Guneser BA. Cold pressed versus solvent extracted lemon (*Citrus limon* L.) seed oils: yield and properties. J Food Sci Technol. (2017) 54:1891–900. 10.1007/s13197-017-2622-8 28720945 PMC5495713

[30] McClements DJ. Colloidal basis of emulsion color. Curr Opin Colloid Interface Sci. (2002) 7:451–5. 10.1016/S1359-0294(02)00075-4 12027025

[31] Arya SS Ramanujam S Vijayaraghavan PK. Refractive index as an objective method for evaluation of rancidity in edible oils and fats. J Am Oil Chem Soc. (1969) 46:28–30. 10.1007/BF02632705

[32] Oyaizu M. Studies on products of browning reaction: anti-oxidative activity of products of browning reaction prepared from glucosamine. Jpn J Nutr. (1986) 44:307–15. 10.5264/eiyogakuzashi.44.307

[33] Benzing-Purdie LM Ripmeester JA Ratcliffe CI. Effects of temperature on Maillard reaction products. J Agric Food Chem. (1985) 33:31–3. 10.1021/jf00061a009

[34] Karseno E Yanto T Setyowati R Haryanti P. Effect of pH and temperature on browning intensity of coconut sugar and its antioxidant activity. Food Res. (2018) 2:32–8.

[35] Houston MC Harper KJ. Potassium, magnesium, and calcium: their role in both the cause and treatment of hypertension. J Clin Hypertens. (2008) 10:3–11. 10.1111/j.1751-7176.2008.08575.x 18607145 PMC8109864

[36] Wang X Yang R Jin X Zhou Y Han Y Gu Z. Distribution of phytic acid and associated catabolic enzymes in soybean sprouts and indoleacetic acid promotion of Zn, Fe, and Ca bioavailability. Food Sci Biotechnol. (2015) 24:2161–7. 10.1007/s10068-015-0288-4

[37] Mohammed NK Manap MYA Tan CP Muhialdin BJ Alhelli AM Hussin ASM. The effects of different extraction methods on antioxidant properties, chemical composition, and thermal behavior of black seed (*Nigella sativa* L.) oil. Evid Based Compl Alter Med. (2016) 2016:6273817. 10.1155/2016/6273817 27642353 PMC5015008

[38] Rice-Evans CA Miller NJ Bolwell PG Bramley PM Pridham JB. The relative antioxidant activities of plant-derived polyphenolic flavonoids. Free Radical Res. (1995) 22:375–83. 10.3109/10715769509145649 7633567

[39] Maksimovic Z Malencic D Kovacevic N. Polyphenol contents and antioxidant activity of Maydis stigma extracts. Bioresour Technol. (2005) 96:873–7. 10.1016/j.biortech.2004.09.006 15627557

[40] Wang YF Wang XY. Binding, stability, and antioxidant activity of quercetin with soy protein isolate particles. Food Chem. (2015) 188:24–9. 10.1016/j.foodchem.2015.04.127 26041159

[41] Reazai M Mohammadpourfard I Nazmara S Jahanbakhsh M Shiri L. Physicochemical characteristics of citrus seed oils from Kerman, Iran. J Lipid. (2014) 2014:174954. 10.1155/2014/174954 25136460 PMC4127231

[42] Mahmud S Akhtar H Saleem M Khanum R. Lipid classes of *in vitro* cultivar of Pakistani citrus. J Saudi Chem Soc. (2009) 13:299–302. 10.1016/j.jscs.2009.10.013

[43] Zárate R el Jaber-Vazdekis N Tejera N Pérez JA Rodríguez C. Significance of long chain polyunsaturated fatty acids in human health. Clin Trans Med. (2017) 6:25. 10.1186/s40169-017-0153-6 28752333 PMC5532176

[44] Hoppenbrouwers T Cvejic-Hogervorst JH Garssen J Wichers HJ Willemsen LE. Long chain polyunsaturated fatty acids (LCPUFAs) in the prevention of food allergy. Front Immunol. (2019) 10:1118. 10.3389/fimmu.2019.01118 31178862 PMC6538765

[45] Lota ML de Rocca Serra D Tomi F Jacquemond C Casanova J. Volatile components of peel and leaf oils of lemon and lime species. J Agric Food Chem. (2002) 50:796–805. 10.1021/jf010924l 11829647

[46] Flamini G Tebano M Cioni PL. Volatiles emission patterns of different plant organs and pollen of *Citrus limon*. Anal Chim Acta. (2007) 589:120–4. 10.1016/j.aca.2007.02.053 17397661

[47] Flamini G Cioni PL. Odour gradients and patterns in volatile emission of different plant parts and developing fruits of grapefruit (*Citrus paradisi* L.). Food Chem. (2010) 120:984–92. 10.1016/j.foodchem.2009.11.037

[48] Cannon RJ Kazimierski A Curto NL Li J Trinnaman L Jańczuk AJ. Identification, synthesis, and characterization of novel sulfur-containing volatile compounds from the in-depth analysis of lisbon lemon peels (*Citrus limon* L. *Burm*. f. cv. *Lisbon*). J Agric Food Chem. (2015) 63:1915–31. 10.1021/jf505177r 25639384

[49] Spadaro F Circosta C Costa R Pizzimenti F Palumbo DR Occhiuto F. Volatile fraction composition and biological activity of lemon oil (*Citrus limon* L. Burm): comparative study of oils extracted from conventionally grown and biological fruits. J Essent Oil Res. (2012) 24:187–93. 10.1080/10412905.2012.659518

[50] Zhang H Xie Y Liu C Chen S Hu S Xie Z. Comprehensive comparative analysis of volatile compounds in citrus fruits of different species. Food Chem. (2017) 230:316–26. 10.1016/j.foodchem.2017.03.040 28407917

[51] González-Mas MC Rambla JL López-Gresa MP Blázquez MA Granell A. Volatile compounds in citrus essential oils: a comprehensive review. Front Plant Sci. (2019) 10:12. 10.3389/fpls.2019.00012 30804951 PMC6370709

[52] Gould MN. Cancer chemoprevention and therapy by monoterpenes. Environ Health Perspect. (1997) 105:977–9. 9255590 10.1289/ehp.97105s4977 PMC1470060

[53] Sun J. D-limonene: safety and clinical applications. Altern Med Rev. (2007) 12:259–64. 18072821

[54] Limonene monograph. Cri Rev Food Sci Nutr. (1999) 39:260–5.

[55] Melero JA Vicente G Morales G Paniagua M Bustamante J. Oxygenated compounds derived from glycerol for biodiesel formulation: influence on EN 14214 quality parameters. Fuel. (2010) 89:2011–8. 10.1016/j.fuel.2010.03.042

[56] Martínez G Sánchez N Encinar JM González JF. Fuel properties of biodiesel from vegetable oils and oil mixtures. Influence of methyl esters distribution. Biomass Bioenerg. (2014) 63:22–32. 10.1016/j.biombioe.2014.01.034

[57] Coronado CR de Carvalho JA Jr Yoshioka JT Silveira JL. Determination of ecological efficiency in internal combustion engines: The use of biodiesel. Appl Therm Eng. (2009) 29:1887–92. 10.1016/j.applthermaleng.2008.10.012

[58] Encinar JM González JF Rodríguez-Reinares A. Biodiesel from used frying oil. Variables affecting the yields and characteristics of the biodiesel. Ind Eng Che Res. (2005) 44:5491–9. 10.1021/ie040214f

[59] Demirbas A. Political, economic and environmental impacts of biofuels: A review. Appl Energy. (2009) 86:S108–17. 10.1016/j.apenergy.2009.04.036

