# Supplementary figures and images for: Quality Characteristics and Antioxidant Potential of Lemon (Citrus limon Burm. f.) Seed Oil Extracted by Different Methods
Source: Front Nutr. 2021 Sep 9;8:644406. doi: 10.3389/fnut.2021.644406 (PMC8458774; doi:10.3389/fnut.2021.644406)

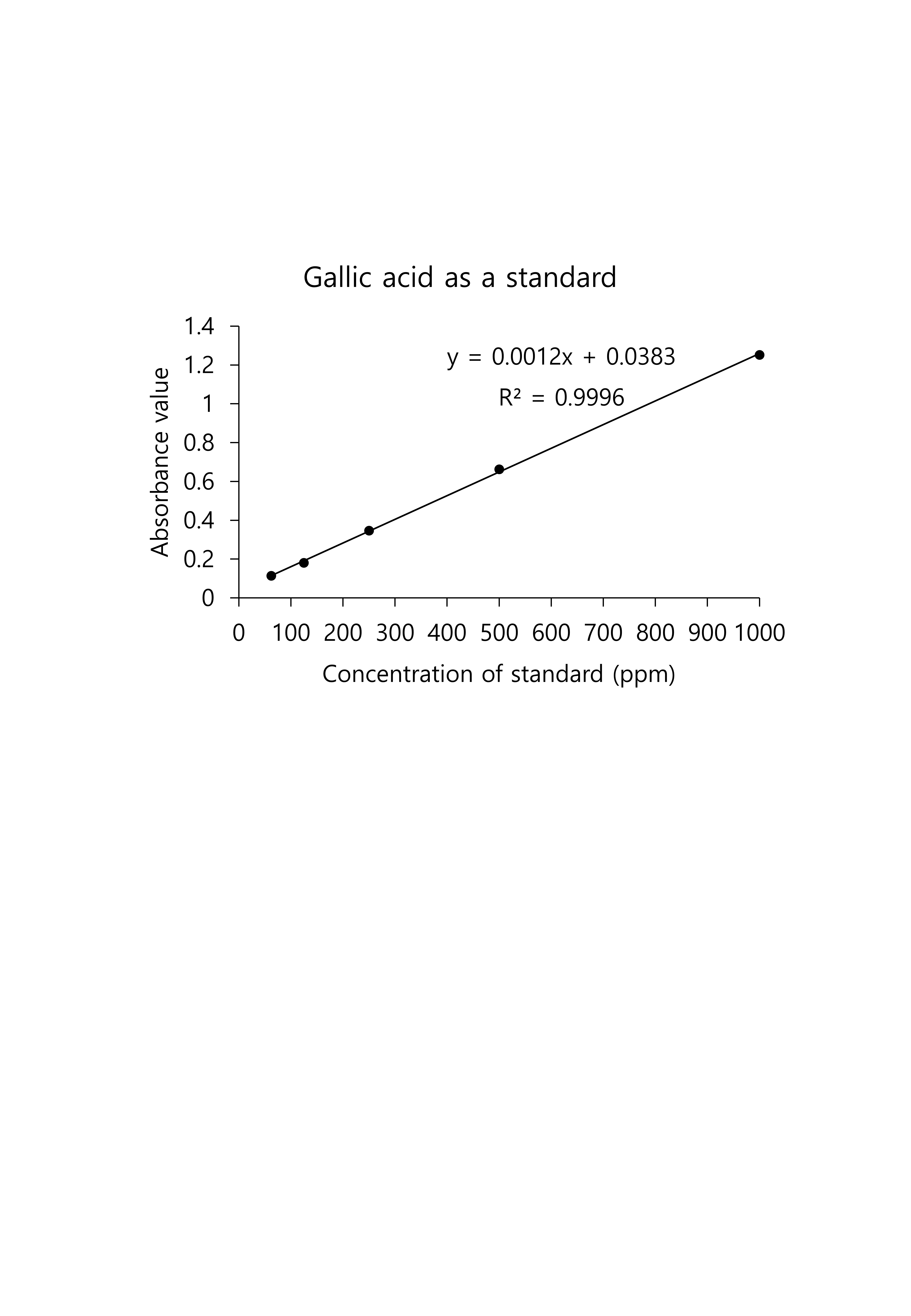

Supplement: Supplementary file 1 [file Image_1.PNG]

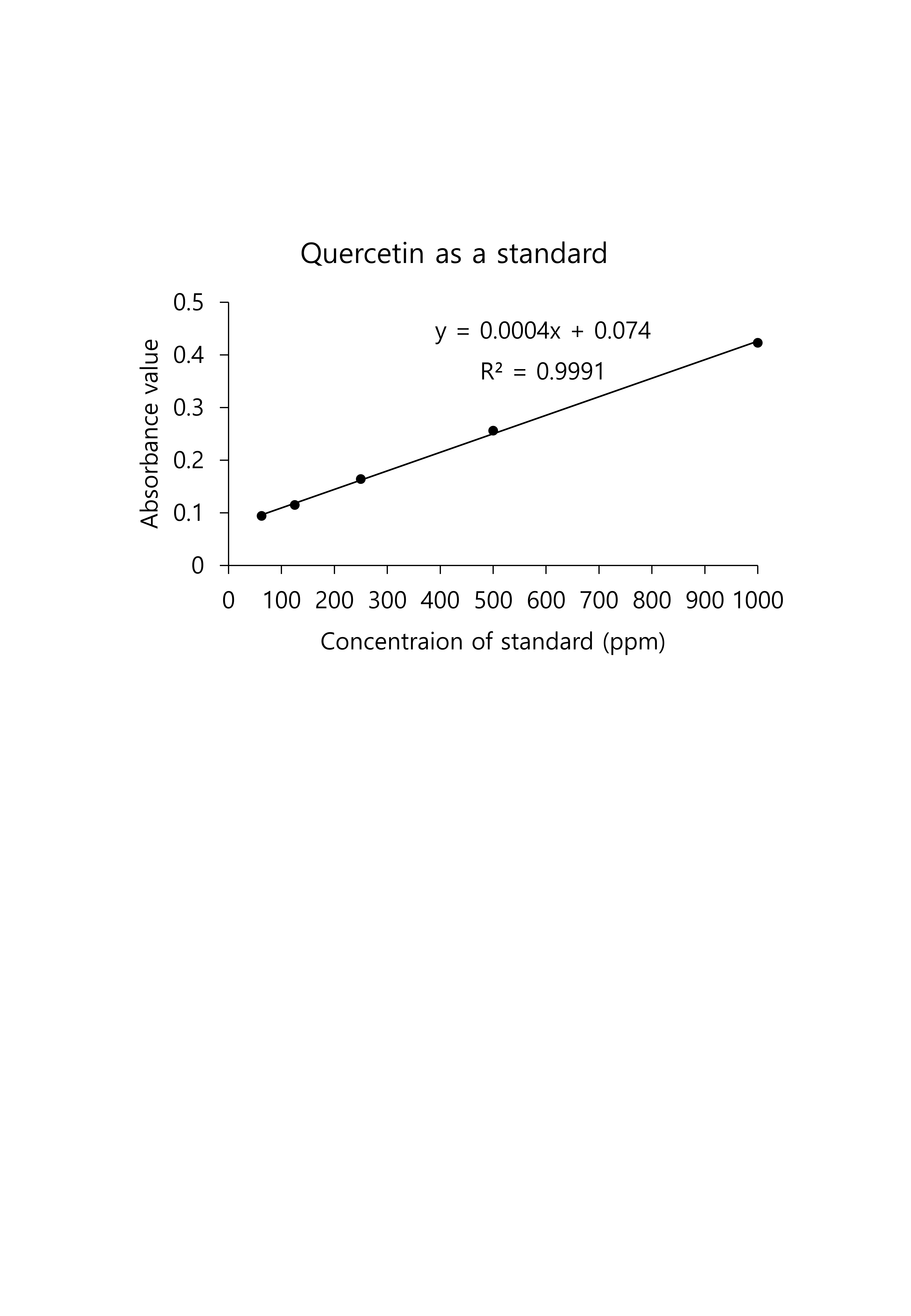

Supplement: Supplementary file 2 [file Image_2.PNG]

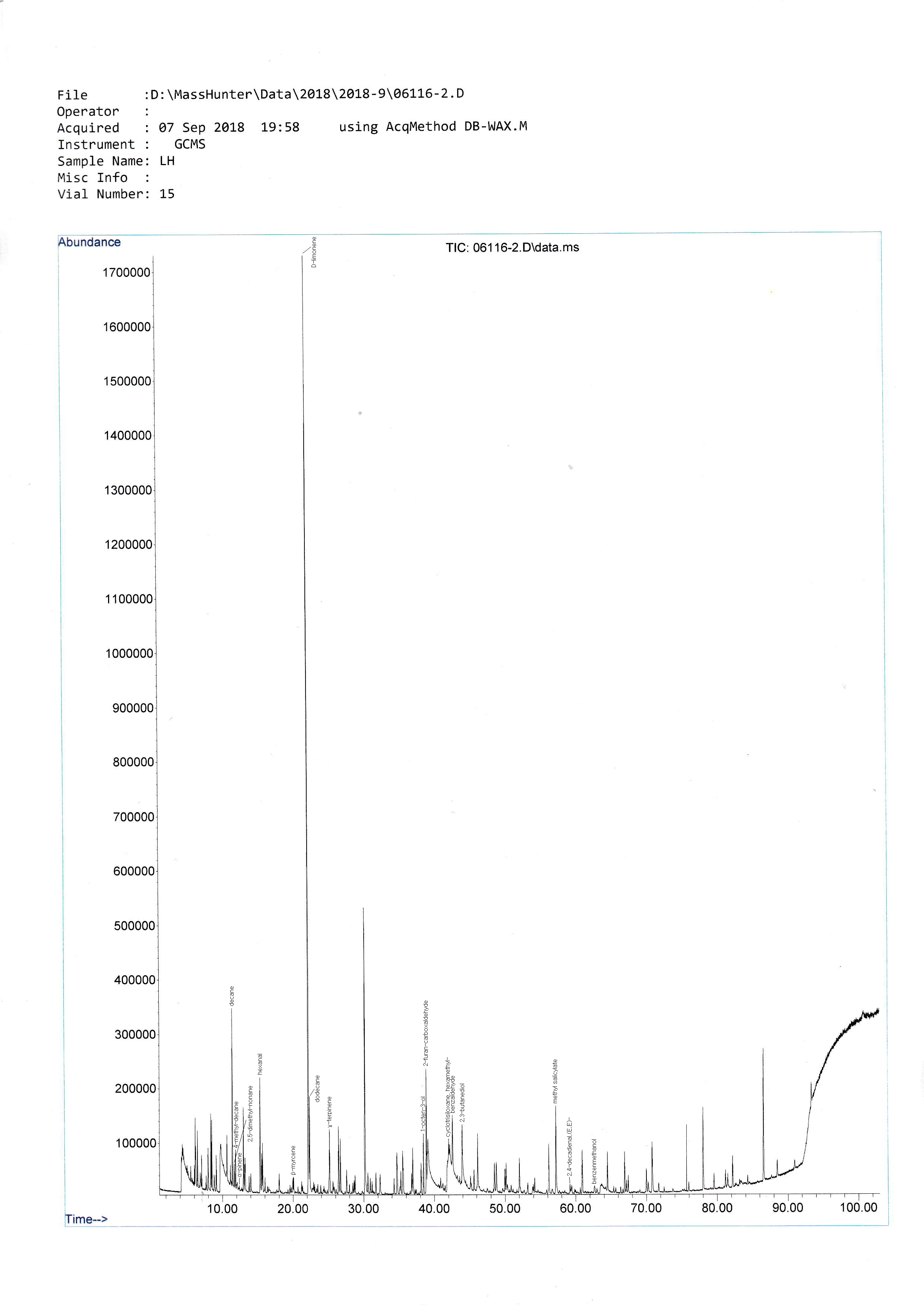

Supplement: Supplementary file 4 [file Image_4.PNG]

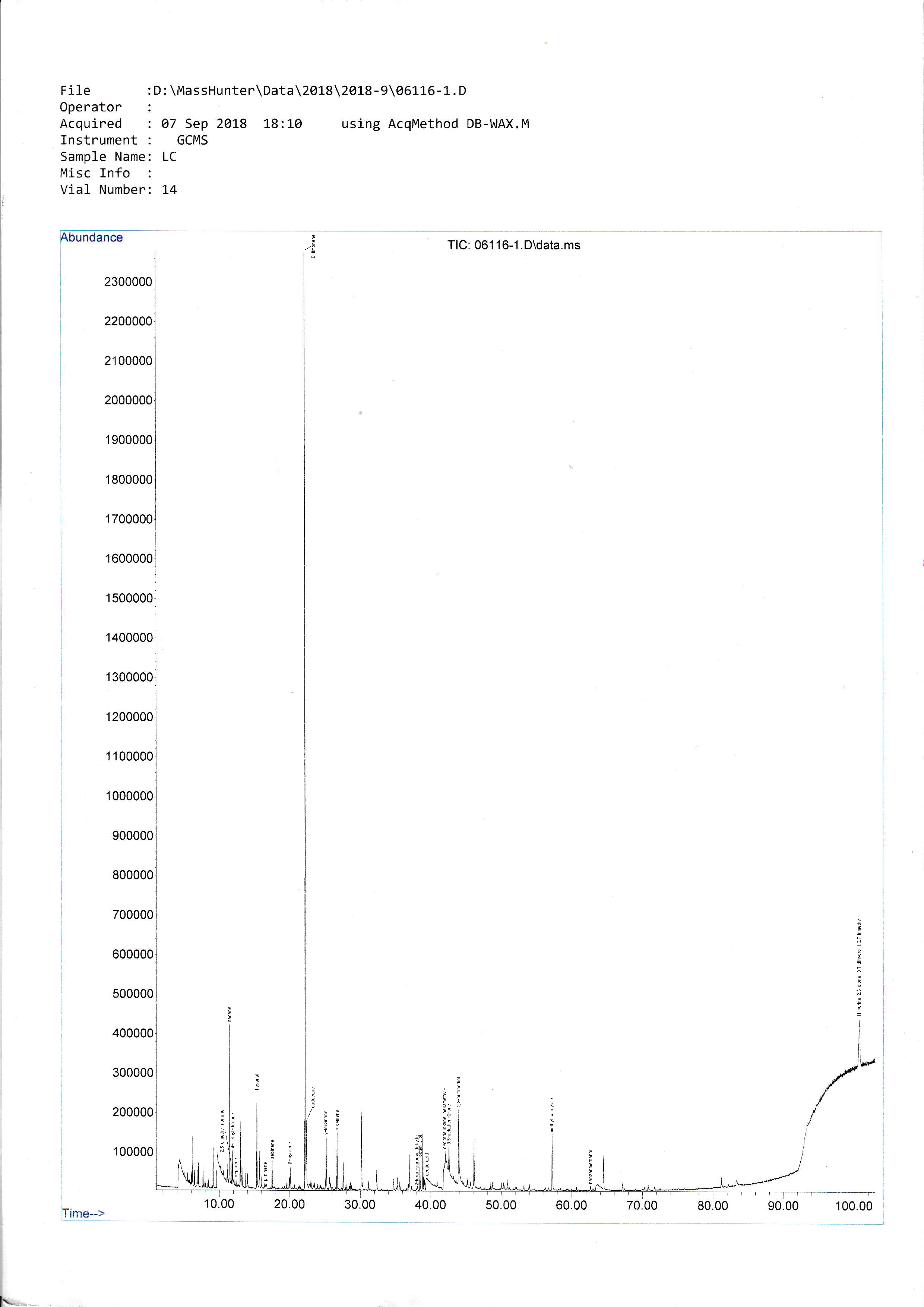

Supplement: Supplementary file 5 [file Image_5.PNG]

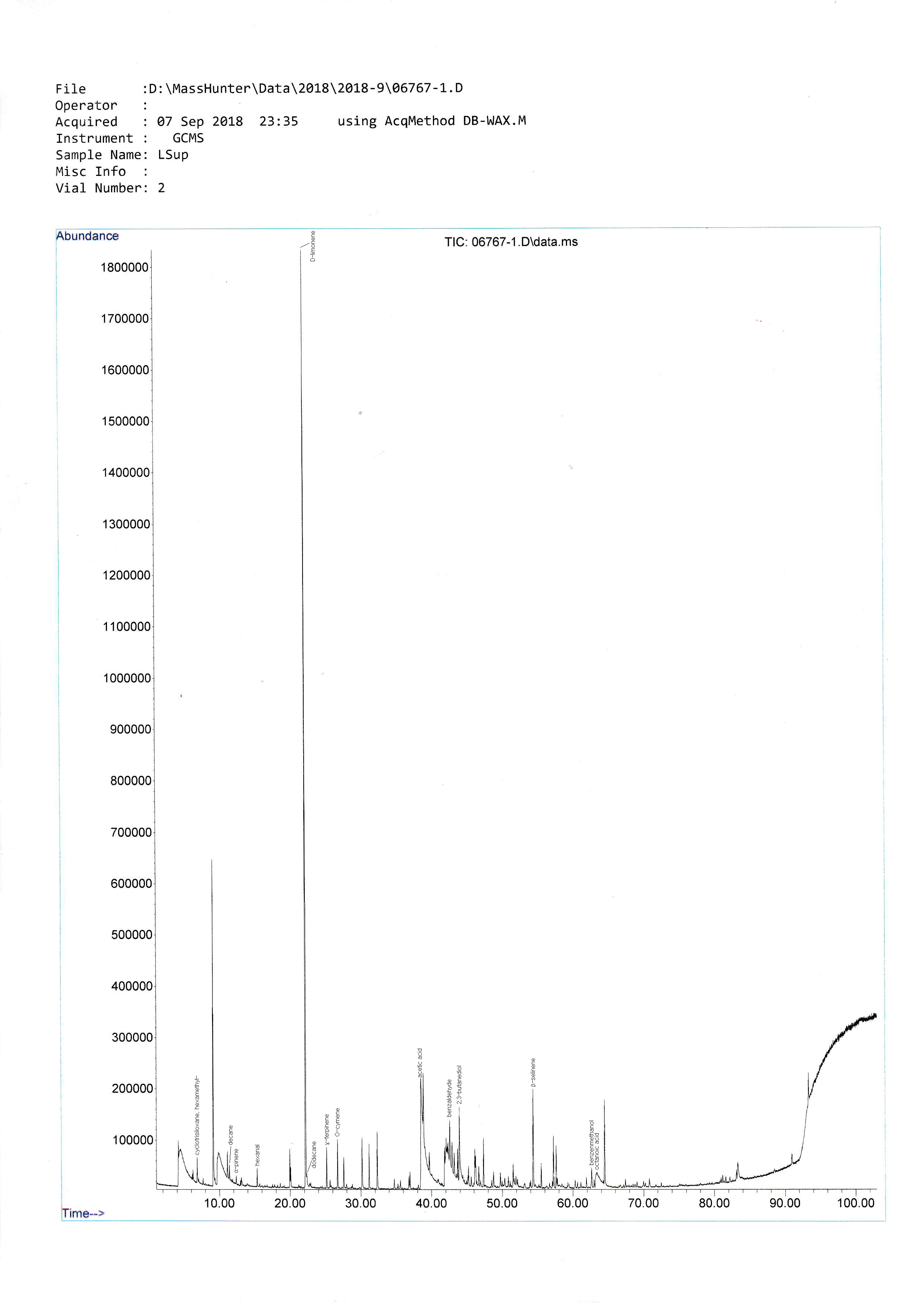

Supplement: Supplementary file 6 [file Image_6.PNG]
